# Two Distinct States of the HAMP Domain from Sensory Rhodopsin Transducer Observed in Unbiased Molecular Dynamics Simulations

**DOI:** 10.1371/journal.pone.0066917

**Published:** 2013-07-02

**Authors:** Ivan Gushchin, Valentin Gordeliy, Sergei Grudinin

**Affiliations:** 1 Institut de Biologie Structurale Jean-Pierre Ebel, Université Joseph Fourier – Grenoble 1, Grenoble, France; 2 Institut de Biologie Structurale Jean-Pierre Ebel, Commissariat à l’énergie atomique, Grenoble, France; 3 Institut de Biologie Structurale Jean-Pierre Ebel, Centre national de la recherche scientifique, Grenoble, France; 4 Research-educational Centre “Bionanophysics”, Moscow Institute of Physics and Technology, Dolgoprudniy, Russia; 5 Institute of Complex Systems (ICS), ICS-6: Structural Biochemistry, Research Centre Jülich, Jülich, Germany; 6 NANO-D, Inria Grenoble-Rhone-Alpes Research Center, Montbonnot, France; 7 Laboratoire Jean Kuntzmann, Centre national de la recherche scientifique, Grenoble, France; Russian Academy of Sciences, Institute for Biological Instrumentation, Russian Federation

## Abstract

HAMP domain is a ubiquitous module of bacterial and archaeal two-component signaling systems. Considerable progress has been made recently in studies of its structure and conformational changes. However, the mechanism of signal transduction through the HAMP domain is not clear. It remains a question whether all the HAMPs have the same mechanism of action and what are the differences between the domains from different protein families. Here, we present the results of unbiased molecular dynamics simulations of the HAMP domain from the archaeal phototaxis signal transducer NpHtrII. Two distinct conformational states of the HAMP domain are observed, that differ in relative position of the helices AS1 and AS2. The longitudinal shift is roughly equal to a half of an α-helix turn, although sometimes it reaches one full turn. The states are closely related to the position of bulky hydrophobic aminoacids at the HAMP domain core. The observed features are in good agreement with recent experimental results and allow us to propose that the states detected in the simulations are the resting state and the signaling state of the NpHtrII HAMP domain. To the best of our knowledge, this is the first observation of the same HAMP domain in different conformations. The simulations also underline the difference between AMBER ff99-SB-ILDN and CHARMM22-CMAP forcefields, as the former favors the resting state and the latter favors the signaling state.

## Introduction

Many microorganisms live in highly variable environments, and as a consequence they have developed sophisticated signaling systems. Many schemes of signal transduction rely on a phosphotransfer reaction and employ two main components: a histidine kinase, whose activity depends on the signal, and a response regulator protein [1]. Some kinases sense the signal themselves (for example, kinases EnvZ and NarX of *Escherichia coli*), meanwhile others are regulated by chemo- and photoreceptors (the CheA kinases that are widespread among bacteria and archaea).

A ubiquitous module of sensory proteins is the HAMP domain, found in histidine kinases, adenylyl cyclases, methyl-accepting chemotaxis proteins and phosphatases (recently reviewed in [2]). The domain was first identified as an amphipathic linker between the transmembrane helices and the signal output domain [3], [4]. Much later, the atomic structure of the HAMP domain part of the thermophile *Archaeoglobus fulgidus* putative protein Af1503 was determined by NMR [5]. The structure revealed that the HAMP domain is organized as a symmetric homodimeric parallel coiled coil. Each protomer has two α-helices, AS1 and AS2, connected by a flexible linker segment. Later, a similar structure was observed in a crystallographic structure of three consecutive HAMP domains from the Aer2 protein of *Pseudomonas aeruginosa* [6]. The arrangement was also verified by biochemical and biophysical methods for a number of other proteins – chemoreceptors Tar and Tsr [7]–[10], aerotaxis protein Aer [11], [12], phototaxis signal transducer HtrII [13]–[16] and sensory histidine kinases EnvZ and NarX [17], [18].

Currently, there are several models of signal transduction through the HAMP domain [2]. The gearbox model posits that the HAMP domain helices switch between the orthodox a-d packing and the unusual x-da packing [5], [19], [20]. Atomic structures of HAMP domain – DHp phosphotransfer domain fusions show that the rotation of the HAMP domain’ helices results in rotation of adjacent helices of DHp [19], [20]. This mechanism explains the signal transduction in receptor histidine kinases, but it is not clear whether it is the case for chemo- and photoreceptors. Alternatively, experimental data reveal that the signal input in chemoreceptors and NarX is a piston-like motion of the transmembrane helix, to which the HAMP domain is connected [21]–[23]. The HAMP domain itself may switch between two conformations [2], [11]. The output was proposed to be coded by the dynamic properties – looser or tighter packing of the HAMP domain’ helices [2], [7], [8], [24].

As for phototactic signal transducers, it was first proposed that the HAMP domain of NpHtrII transduces the signal via switching between a compact and a highly dynamic states [14], [15]. Later, the fluorescent labeling studies revealed that the helices AS1 and AS2 move in opposite directions during signal transduction [16]. Molecular modeling and NMR studies have shown that the NpHtrII HAMP domains have the same fold as the HAMP domains for which the structure is known [25]–[27].

Recently, several groups have studied the properties of chemo- and phototaxis proteins by means of modeling. Models of the NpHtrII HAMP1 as well as the HAMP domain region were built by our team and Nishikata *et al.* [24], [25]. Nishikata *et al*. have also studied the dynamics of the NpSRII-NpHtrII complex in the ground and the M states by means of molecular dynamics [28]. Signal transduction via the transmembrane part of chemoreceptors Tar [29], [30] and sensor kinase PhoQ [31] was studied extensively by different groups. Finally, Hall *et al*. have generated a model of the entire chemoreceptor Tsr and of the trimer-of-dimers of these chemoreceptors that has shown how the small structural changes may be propagated across the system [31].

Here, we present the results of unbiased molecular dynamics simulations of the first HAMP domain from NpHtrII. We observe two distinct conformational states. Relative positions of the helices AS1 and AS2 in these states differ by as much as half of an α-helix turn. These and others conformational changes are in accord with recent experimental results [2], [6], [16], and thus we propose that the observed states are the resting and the signaling states. To the best of our knowledge, these are the first structures of the same HAMP domain in different conformations.

## Results and Discussion

### Molecular Dynamics Simulations of the HAMP Domain

We have performed the molecular dynamics study of the HAMP domain from halobacterial phototactic signal transducer NpHtrII, the first one of the two HAMP domains present in the protein. The simulations consist of 28 trajectories, each lasting more than 205 ns. The total length of the simulations is more than 6.0 µs. Details of the simulations are presented in the Table 1. Overall, the domain was not changing its fold during the simulations. The average RMSD of the backbone atoms N, C, C_α_, O for the whole simulation is 1.35±0.33 Å. The observed structure of the domain was quite similar to that reported previously [25], [26].

**Table 1:** Details of the performed simulations of the first HAMP domain of NpHtrII.

Simulation #	Starting coordinates	Forcefield	Number of trajectories andtheir length	Average RMSD ofbackbone atoms
1	Symmetrical homology model basedon the Af1503 HAMP domain	CHARMM22 withCMAP correction	10×∼205 ns	1.1 Å
2	Symmetrical homology model basedon the Af1503 HAMP domain	AMBER ff99-SB-ILDN	10×∼205 ns	1.3 Å
3	“Resting state” conformationsfrom different trajectoriesof simulation #1	CHARMM22 withCMAP correction	5×∼205 ns	1.2 Å
4	“Active state” conformationsfrom different trajectoriesof simulation #1	AMBER ff99-SB-ILDN	3×∼205 ns	1.4 Å

We started the simulations from the homology model based on the NMR structure of the Af1503 HAMP domain. Preliminary results have shown that for optimal simulation several factors have to be taken into account. First, the CHARMM22-CMAP and AMBER ff99-SB-ILDN forcefields bias the structure in different ways and thus using only one forcefield is not reliable. We conducted the simulations using both forcefields. Second, the trajectories are highly sensitive to the starting structure, which is the equilibrated homology model. As the temperature and pressure equilibration process is non-deterministic, we calculated several trajectories for each forcefield with the model independently equilibrated each time. This resulted in a good sampling of the HAMP domain conformational space as each starting structure had a RMSD of backbone atoms’ positions of ∼0.3 Å relative to the energy-minimized structure and of ∼0.5 Å relative to the other starting structures. Finally, the N- and C-termini of the HAMP domain were unfolding on the scale of 20–100 ns. As we expect this to be a result of non-native truncation of the model, the α-helical structure of 4 residues from each terminus was restrained.

### Two States of the NpHtrII HAMP Domain

**Figure 1:**
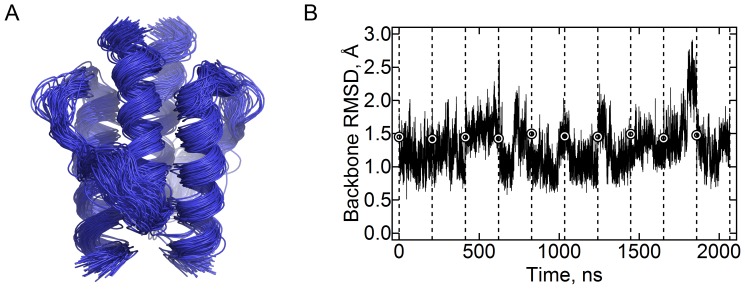
Stability of the HAMP domain in molecular dynamics simulations. (A) Superposition of the conformations observed in the simulations with CHARMM22-CMAP forcefield. The snapshots taken each 20 ns are shown. The domain remains stable during the simulations. (B) Root-mean square deviation of the backbone atom positions relative to the average observed in the simulations with CHARMM22-CMAP forcefield. The mean value is 1.3 Å. The data for ten trajectories are shown consecutively; they are separated by the dashed lines. The circles denote the value at the start of each trajectory.

**Figure 2:**
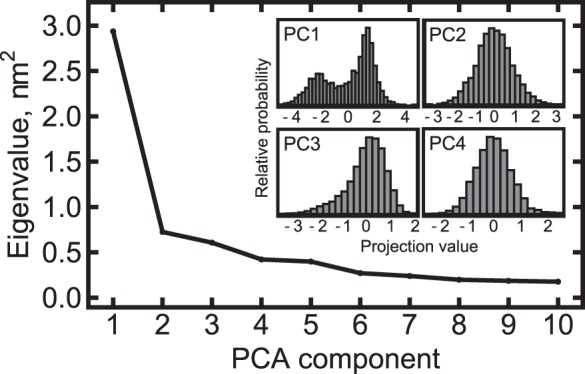
Principal components analysis of the calculated trajectories. The covariation matrix is dominated by the first principal component (PC1) that accounts for 35% of the matrix trace. Histograms of the trajectory projections on the corresponding principal components are shown in the inset. For PC1, the distribution is bimodal, and for the other principal components it is unimodal. From that, we conclude that PC1 corresponds to transitions between two distinct states, and the other components reflect thermal fluctuations.

Initial simulations #1 and #2 (Table 1) with the forcefields CHARMM22-CMAP and AMBER ff99-SB-ILDN revealed significant motions of the HAMP domain’s helices (data for the CHARMM forcefield is presented in Figure 1A; data for the Amber forcefield are similar), despite the relatively low overall RMSD values (Figure 1B; data for the AMBER forcefield are similar). Two metastable states were discernible by visual examination. To obtain quantitative measures, we employed the principal components analysis [33]. The analysis revealed that the motions are dominated by the first principal component (PC1, 35% of total variation, meanwhile the second and third components account for 8.5% and 7% correspondingly), for which the distribution of the projections is bimodal; the distribution is unimodal for other principal components (Figure 2).

**Figure 3:**
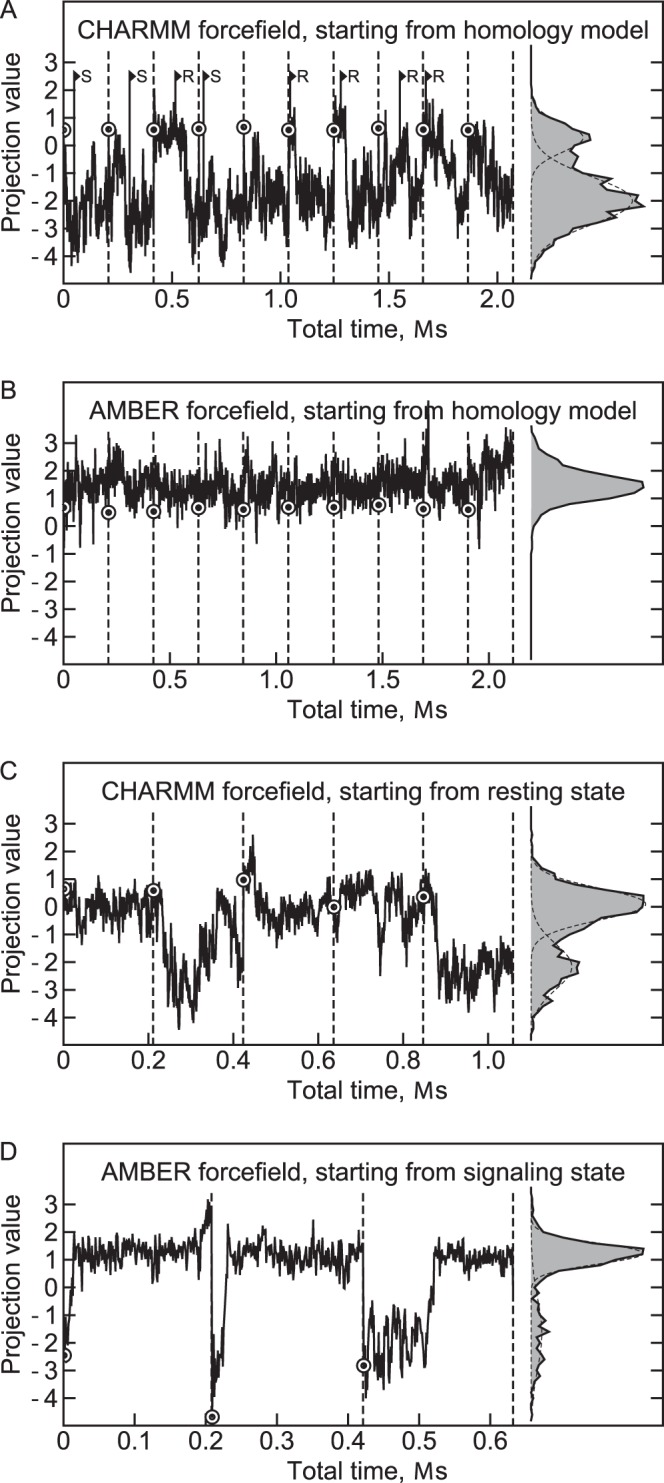
Projections of the trajectories on the first principal component. Panels A, B, C and D show the data for the simulations #1, #2, #3 and #4 correspondingly. The projections follow bimodal distribution with centers at approximately 0.2 and −2.1 for the CHARMM simulations and 1.3 and −1.7 for the AMBER simulations (fitted by Gaussian distributions). Higher projection values correspond to the resting state and lower values correspond to the signaling state. The CHARMM forcefield favors the signaling state, and the AMBER forcefield favors the resting state. The data for different trajectories are shown consecutively and are separated by the dashed lines. The circles denote the value at the start of each trajectory. The R (resting) and S (signaling) signs at the panel A denote the starting frames for the simulations #3 and #4.

There were differences between the simulations performed using the CHARMM and the AMBER forcefield (Figure 3A,B). Meanwhile in the AMBER simulation the projections on the first principal component group around the value of ∼1.3, the projections in the CHARMM simulation follow a bimodal distribution with centers at ∼0.2 and −2.1. AMBER favors the values in the range 1÷2 and CHARMM favors the values in the range −3÷−1. In some of the CHARMM trajectories, the domain immediately switched to the negative projections (trajectories 4, 5 and 8), meanwhile in others it stayed close to the initial structure for some time (trajectories 3, 6 and 7). Some trajectories revisited the starting state.

We were interested to determine whether there are really two distinctive states (with the average projections of 0.2÷1.3 and −2.1) or this is a result of a forcefield bias. To that end, we have performed additional simulations #3 and #4, with the structures from the simulation #1 taken as starting poses (Figure 3A). In the CHARMM forcefield simulations, the starting structures had initial projection values in the range 0÷1, and in the AMBER forcefield simulations, the starting structures had initial projection values in the range −4÷−2 (Figure 3C,D, Table 1). In both simulations, the domain spent at least some time in the starting state. Transitions from the starting state to the forcefield-preferred state were observed in both simulations and occurred in a switch-like fashion (Figure 3C,D). Thus, we conclude that each forcefield recognizes two distinct states. At the same time, CHARMM favors the state with the average projection of −2.1 meanwhile AMBER clearly favors the other state with projections in the range of 0.2÷1.3.

**Figure 4:**
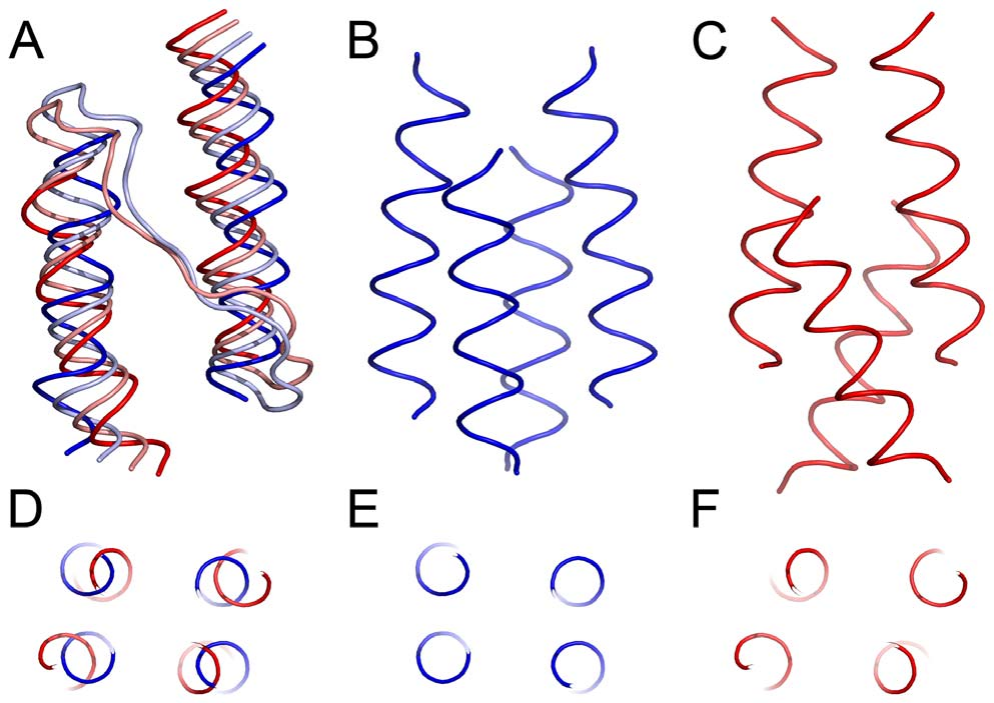
Conformational changes associated with the first principal component. (A), (B) and (C) Side view of the HAMP domain. (D), (E) and (F) View along the HAMP domain axis. The extreme projections are shown in blue (resting state) and red (signaling state), and the state averages are shown in light blue and pink correspondingly (A). In the resting state (B, E), the alpha-helices AS1 and AS2 are more parallel and the HAMP domain cross-section is rectangular. In the signaling state (C, F), AS1 and AS2 are displaced in opposite directions along the HAMP domain axis, are no longer parallel and the cross-section is rhombic. The extreme projections are shown as they illustrate best the conformational changes involved.

**Figure 5:**
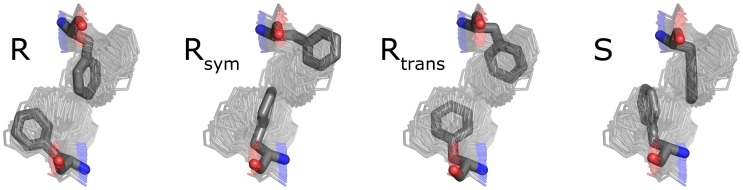
Configurations of F124 pair in the resting and signaling states. Representatives of each configuration are shown. For reference, the structures are highlighted in the ensemble of snapshots taken each 10 ns and aligned by the C, Ca, N and O atoms of F124. R and R_sym_ correspond to the resting state and are basically the same state as they are related by 180° rotation around the HAMP domain axis. R_trans_ is a transitional conformation between R and R_sym_, observed transiently in AMBER simulations. S is the conformation observed in the signaling state.

**Figure 6:**
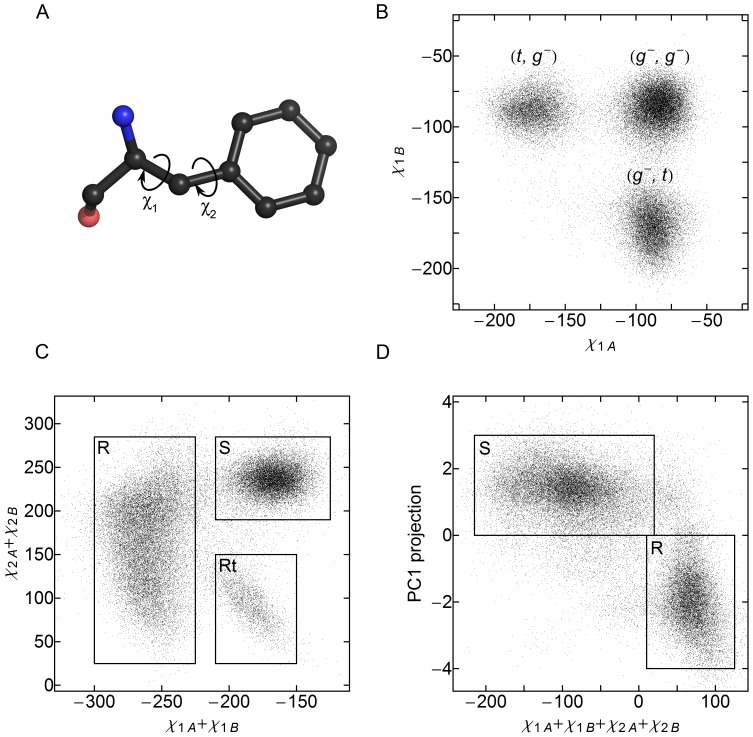
Use of F124 χ_1_ and χ_2_ dihedral angles to classify the HAMP domain state. Subscripts A and B denote the protomers of the dimeric HAMP domain. (A) Definition of the *χ_1_* and *χ_2_* dihedral angles of phenylalanine sidechain. (B) Distribution of the *χ_1_* angles during the simulations. *t* stands for the *trans* conformation, and *g^-^* for the *gauche^-^* conformation. (*t*, *t*) conformation is not observed. (C) Plot of the sum of the *χ_2_* angles as a function of the sum of the *χ_1_* angles. There are two substates corresponding to *χ_1_* (*g^-^*, *g^-^*) conformation (*χ_1_* sum of ∼-170°). The state with the *χ_2_* sum of ∼240° corresponds to the signaling state, meanwhile the state with the sum of ∼100° is transient and visited during transitions from the *χ_1_* (*t*, *g^-^*) conformation to (*g^-^*, *t*). (D) Relation between the F124 conformations, represented by the sum of side chain dihedral angles, and the whole domain backbone conformation, represented by the value of the projection on the first principal component. Unambiguous correspondence may be established between these two values.

**Figure 7:**
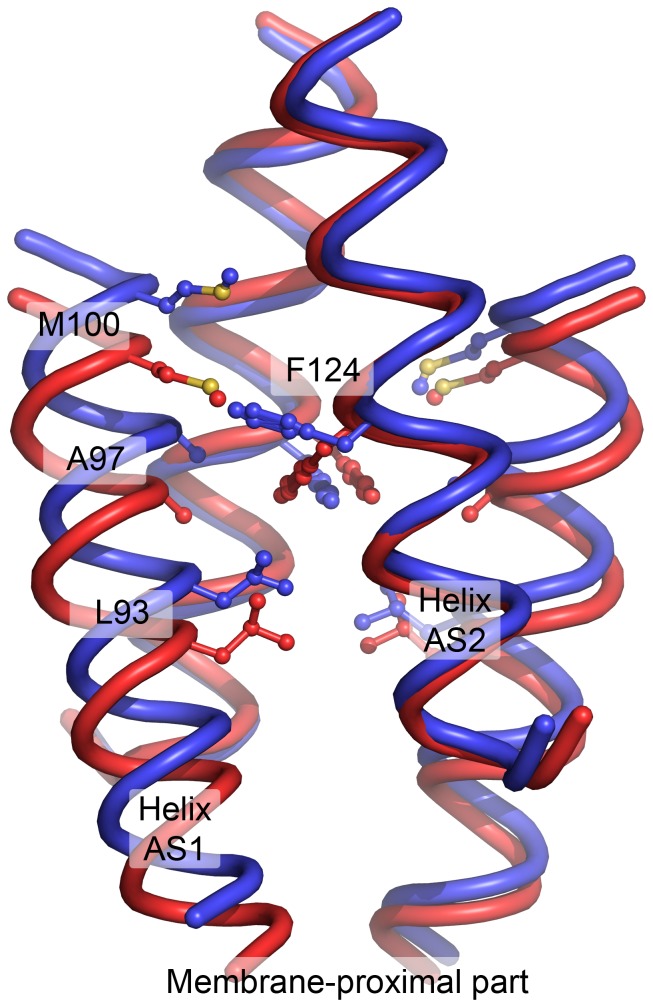
Comparison of the resting (blue) and the signaling (red) states of the NpHtrII HAMP1 domain obtained in molecular dynamics simulations. Conformation of the F124 pair is closely related to the conformation of the whole HAMP domain. The averaged structures from the CHARMM simulation are shown. For the resting state, only the structures with similar F124 position were chosen and not the symmetrically related ones, that is only (*t*, *g^-^*) and not (*g^-^*, *t*). The most notable difference between the observed states is a longitudinal displacement of the AS1 helices relative to the AS2 helices. Following the coiled-coil terminology, F124 from the helix AS2 forms a “knob” in the “hole” made of the AS1 residues L93, A97 and M100 of the same protomer. These residues are shown in sticks representation. As a consequence of the interaction, position of AS1 relative to AS2 is closely linked to the F124 rotameric state. Also, in the signaling state, F124 bulges less and the helices are more mobile. The averaged structure of the signaling state is symmetrical. The resting state is inherently asymmetric due to F124, however the backbone atoms’ positions in R and Rsym states are almost identical in the AMBER simulations, and are similar in the CHARMM simulations.

The eigenvector corresponding to PC1 characterizes the structural details of the transition between the two discovered states. To determine it in the best way possible we have applied the analysis to the concatenated trajectory that includes all the simulations. The same PC1 was used to obtain all the results presented here (Figures 2–7). Comparison of the eigenvectors obtained from individual simulations with those determined from the concatenated trajectory is shown in Figure S1. The PC1 determined from the concatenated trajectory has no analogs in the simulation #2, as the second state and the state transition are not present in the latter. In the other simulations, the principal component 1 is highly similar to the one determined from the concatenated trajectory, with the dot products of their normalized eigenvectors being 0.93, 0.77 and 0.95 for simulations #1, #3 and #4 correspondingly. Dot products of PC1 eigenvectors of simulations #1 and #3 as compared to #4 are 0.91 and 0.74.

It could be useful to estimate the free energy difference and the value of the energy barrier between the two states. However, the results obtained with the AMBER and CHARMM forcefields differ not only in the magnitude of the energy difference but also in its sign. Inclusion of the adjacent NpHtrII domains into the model might also change the energetics of the transition from one state to another. Thus, we do not assess the aforementioned energetic properties as the results of such assessment may be misleading. The presented simulations may serve as an example that, despite considerable progress in forcefield development [34], [35], a choice of a forcefield can still affect the results, not only quantitatively but also qualitatively.

### Structural Analysis of the Two Observed States

After establishment of the two states of the NpHtrII HAMP domain we proceed to the structural analysis. The most notable conformational rearrangement associated with the first principal component is the relative longitudinal displacement of the HAMP domain helices AS1 and AS2 (Figure 4A,B,C and Movies S1 and S2). Such longitudinal displacement of the NpHtrII HAMP1 helices was recently observed experimentally [16]: the HAMP domain was more compact in the resting state and more elongated in the signaling state. Given this, we propose that the observed two states correspond to the resting (projection values 0÷2) and the signaling (projection values −3÷−1) states.

Another notable feature is the change in the cross-section shape of the HAMP domain (Figure 4D,E,F and Movie S1). In the resting state, the helices are arranged rectangularly, and in the signaling state they are arranged rhombically. This is similar to the compact and loose conformations of the HAMP domains observed in the crystallographic structures of the Aer2 aerotaxis transducer [2], [11].

The conformations corresponding to the two states are almost symmetrical (Figure 4B,C). However, thorough analysis has revealed that in the resting state the positions of the core hydrophobic residues F124 are not symmetric. Thus, the symmetry of the resting state structure presented in Figure 4 is a result of mixing of two asymmetric structures.

Asymmetry has already been noted in other simulations of the HAMP-domain containing systems [25]. It also has been noted that the NpHtrII HAMP1 possesses bulky hydrophobic aminoacids at its core [26] and the bulkiness of the insidious residues is crucial for the HAMP domain function [5], [19], [20]. To determine the correct asymmetric resting state, we proceeded to analyze the conformations of the F124 pair in different states of the HAMP domain.

### Conformation of the F124 Pair at the HAMP Domain Core

Pair of the F124 residues, residing at the HAMP domain core, adopts several conformations: symmetric S in the signaling state, asymmetric R and R_sym_ in the resting state and, finally, transitional R_trans_ on the way from R to R_sym_ and back (Figure 5 and Movie S2). We analyzed the distributions of the *χ_1_* and *χ_2_* dihedral angles of F124 sidechain (Figure 6). In accord with the observations, the *χ_1_* angles may be in the (*gauche^-^*, *gauche^-^*), (*gauche^-^*, *trans*) or (*trans*, *gauche^-^*) conformation (Figure 6B). However, analysis of the *χ_1_* angles alone does not allow us to differentiate between the Rtrans and S state of the F124 pair. Plotting the *χ_2_* and *χ_2_’* sum as a function of *χ_1_* and *χ_1_’* sum allows to do that, as the Rtrans and S differ by 90° rotation of the benzene rings of both F124 residues (Figure 6C). Consequently, one can classify the state of the HAMP domain by looking at the sum of all the dihedral angles *χ_1_*, *χ_1_’*, *χ_2_* and *χ_2_’*: the values in the range 15°÷125° correspond to the resting state, meanwhile the values in the range −215°÷15° reflect the signaling state.

It is interesting to compare the classification by F124 dihedral angles with the classification by the whole domain backbone conformation (value of PC1 projection), as both numbers can be determined for each trajectory snapshot. The analysis shows that the correspondence is clear and unambiguous (Figure 6D). We use this fact to determine the structure of the resting state without averaging over R and R_sym_.

### Comparison of the NpHtrII HAMP1 Resting and Signaling States

The most significant difference between the resting and the signaling states of the NpHtrII HAMP1 domain is the longitudinal shift of the helices AS1 and AS1’ relative to the helices AS2 and AS2’ (Figure 7 and Movie S2) upon the transition. In the signaling state, the HAMP domain backbone and its hydrophobic core are symmetric. In the resting state, the symmetry of both the backbone and the core breaks down. Position of the helices AS2 remains roughly symmetric, meanwhile AS1 and AS1’ shift longitudinally, but for a different distance. Following the coiled-coil terminology, F124 of the helix AS2 forms a “knob” in the “hole” made of the AS1 residues L93, A97 and M100 of the same protomer, and thus its conformation is linked to the relative shift of AS1 and AS2 (Figure 7 and Movie S2).

Interestingly, the residue F124 resides in the same coiled coil layer as the residue A291 of the Af1503 HAMP domain. Mutations of A291 to bulkier hydrophobic aminoacids render the domain, which is natively dysfunctional, able to conduct the signal in Taz (Tar-HAMP-EnvZ) chimeras [19] as well as other systems [5], [20]. Taken together with the results of our simulations, these data show the role of large aminoacid side chains at the HAMP domain core.

### Conclusions

Here, we have presented the results of unbiased molecular dynamics simulations of the NpHtrII HAMP1 domain. The domain adopts two conformations that share many features with the two HAMP domain states observed experimentally. To the best of our knowledge, this is the first example where the atomic structures of two different conformations of the same HAMP domain are presented.

Although the simulations of the HAMP domain without the flanking modules may be misleading [25], and the structure of its N- and C-termini could differ if other NpHtrII domains were included in the simulation, comparison of the results with experimental data is in favor of biological relevance of the two observed states. At the moment, it is not clear how a transition between these states could result in a signal transduction via the HAMP domain. The structures suggest that the possible mechanisms are the overall elongation of the HAMP1 or twisting of its helices, resulting in rotation of the output modules relative to the input modules around the dimer axis.

## Methods

### Initial Model Preparation

In all the simulations we have studied the first HAMP domain of *Natronomonas pharaonis* HtrII (residues 85–133). The initial model was obtained by automated homology modeling procedure by SWISS-MODEL server [36] using a HAMP domain from putative protein Af1503, PDB ID 2ASW [5] as a template, similarly to what was described previously [25], [26]. The Af1503 HAMP domain has currently the closest sequence to the NpHtrII HAMP1 among the HAMP domains of known structure. For the helices AS1 and AS2, backbone structure was not changed, whereas the sidechains were mutated to the correct ones. The inter-helical linker of NpHtrII HAMP1 is shorter by one residue than that of Af1503 HAMP, and thus it could not be modeled without introducing structural perturbations. However, the modeled conformation is highly similar to the one observed in previous simulations [25], [26], with hydrophobic residues V107 and L109 facing towards the hydrophobic core of the coiled coil. The modeled protomers were aligned by least-squares method to the dimeric HAMP domain structure PDB ID 2ASW. The resulting homodimer was perfectly symmetrical and was used as a starting structure in molecular dynamics simulations.

### Molecular Dynamics Simulations

Molecular dynamics simulations were conducted using the GROMACS software version 4.5.3 [37] with forcefields CHARMM22 [38] with CMAP correction [39] and AMBER ff99-SB-ILDN [40], [41] (Table 1). The TIP3P model water model was used. All the simulations were run identically. First of all, the initial model was solvated in a water box of approximately 3400 water molecules with NaCl concentration of 1 M. This salt concentration reflects the high salinity of the environment *Natronomonas pharaonis* lives in. The box size was chosen so that the minimal distance between the atoms of periodic images of the protein was 1.6 nm. The resulting system was minimized with a steepest descent algorithm using a tolerance of 1,000 kJ mol^−1^ nm^−1^ and a step size of 0.01 nm. After that, the solvent was equilibrated for 10 ps at a constant volume (NVT ensemble) and then for 10 ps at a constant pressure (NPT ensemble). The protein atoms were harmonically restrained during the solvent equilibration. The starting frames for the simulations #3 and #4 were chosen randomly from those of the simulation #1 with a needed HAMP domain state and are shown in Figure 3A. These simulations were preceded by several (less than 10) energy minimization steps to allow for the forcefield difference. The step size was 2 fs. The Coulombic interactions, van der Waals interactions and the short-range neighborlist were cut off at 1 nm. Electrostatics was calculated using a fourth-order particle mesh Ewald method [37] with a Fourier spacing of 0.16 nm. The temperature was kept at 310 K using the modified Berendsen thermostat [42] with a time constant of 1 ps. The pressure was kept equal to 1 bar using the Parrinello-Rahman scheme [43] with a time constant of 1 ps and a compressibility of 4.5×10^−5^ bar^−1^.

To restrain alpha-helicity, harmonic potentials were applied to the φ and ψ dihedral backbone angles. The average values of −63.8° and −41.1° [44] correspondingly were used. The rigidity constant was chosen so that the 10°-deviation corresponded to the energy penalty of 1 kT (at 310 K). For the production runs, 4 residues at the N-terminus and 4 residues at the C-terminus were restrained. Dihedral backbone angles of other AS1 or AS2 residues were not restrained.

### Principal Components Analysis

Principal components analysis (PCA) [33] was conducted using the tools g_covar and g_anaeig of the GROMACS suite [37]. For the analysis, we used the coordinates of heavy backbone atoms (N, C, C_α_, O) of the HAMP domain alpha-helices. Atoms of the linker were not included in the analysis as the linker was very flexible and adopted variable conformations that correlated weakly with the state of the alpha-helices. The principal component 1 determined for the concatenated trajectory that includes all the simulations was used throughout the analysis. Comparison of the principal components determined for individual simulations with those determined for the concatenated trajectory is presented in Figure S1. For the analysis of the F124 motions, values of the backbone PC1 projection and F124 dihedral angles were independently determined for each trajectory snapshot.

## Supporting Information

Figure S1 **Comparison of the principal components obtained from the individual simulations with those obtained from the concatenated trajectory.** The plots show the dot products of the corresponding principal components, with the black color corresponding to 1 and the white color corresponding to 0. The first 20 components were taken for each trajectory. The eigenvectors were normalized to 1 prior to multiplication. The first principal component (PC1), obtained from the concatenated trajectory, is present in both CHARMM simulations (A and C), absent from the AMBER simulation that starts from the homology model (B), but present in the AMBER simulation that starts from the signaling state, obtained previously in the CHARMM simulation. Roughly diagonal structure is observed in all plots. The principal components other than the first one are not very well conserved in different trajectories.
(EPS)

Movie S1 **Transformation of the HAMP domain structure corresponding to the first principal component of the PCA analysis.** The structure is colored blue in the resting-like states and red in the signaling-like states.
(MP4)

Movie S2 **Example of the signaling-to-resting state transition from the molecular dynamics simulations.** Presented is a part of the trajectory 3 from the simulation 4 (AMBER forcefield, starting from the signaling state). The arrows highlight the motions of the helices AS1 and AS1’. The trajectory is smoothened with a window of 11 ns in order to remove fast motions. Projection of the structure on the first principal component is also shown.
(MP4)
